# In a large‐volume multidisciplinary setting individual surgeon volume does not impact LVAD outcomes

**DOI:** 10.1111/jocs.16783

**Published:** 2022-07-21

**Authors:** Joel C. Boudreaux, Marian Urban, Anthony W. Castleberry, John Y. Um, Michael J. Moulton, Aleem Siddique

**Affiliations:** ^1^ College of Medicine University of Nebraska Medical Center Omaha Nebraska USA; ^2^ Department of Surgery University of Nebraska Medical Center Omaha Nebraska USA

**Keywords:** circulatory support, heart failure, ventricular assist device

## Abstract

**Background:**

In complex operations surgeon volume may impact outcomes. We sought to understand if individual surgeon volume affects left ventricular assist device (LVAD) outcomes.

**Methods:**

We reviewed primary LVAD implants at an experienced ventricular assist devices (VAD)/transplant center between 2013 and 2019. Cases were dichotomized into a high‐volume group (surgeons averaging 11 or more LVAD cases per year), and a low‐volume group (10 or less per year). Propensity score matching was performed. Survival to discharge, 1‐year survival, and incidence of major adverse events were compared between the low‐ and high‐volume groups. Predictors of survival were identified with multivariate analysis.

**Results:**

There were 315 patients who met inclusion criteria‐45 in the low‐volume group, 270 in the high‐volume group. There was no difference in survival to hospital discharge between the low (91.9%) and high (83.3%) volume matched groups (*p* = .22). Survival at 1‐year was also similar (85.4% vs. 80.6%, *p* = .55). There was no difference in the incidence of major adverse events between the groups. Predictors of mortality in the first year included: age (hazards ratio [HR]: 1.061, *p* < .001), prior sternotomy (HR: 1.991, *p* = .01), increasing international normalized ratio (HR: 4.748, *p* < .001), increasing AST (HR: 1.001, *p* < .001*)*, increasing bilirubin (HR: 1.081, *p* = .01), and preoperative mechanical ventilation (HR: 2.662, *p* = .005). Individual surgeon volume was not an independent predictor of discharge or 1‐year survival.

**Conclusion:**

There was no difference in survival or adverse events between high and low volume surgeons suggesting that, in an experienced multidisciplinary setting, low‐volume VAD surgeons can achieve similar outcomes to their high‐volume colleagues.

## 1 INTRODUCTION

Ventricular assist devices (VADs) are a widely available treatment option for patients with advanced heart failure, with more than 3100 devices being implanted in the United States in 2019.^1^ Risk factors for mortality and adverse events following left ventricular assist device (LVAD) implantation, including patient factors, device selection, and procedural‐related factors, have been studied extensively.^1^, ^2^, ^3^, ^4^ However, while a variety of studies have demonstrated a relationship between surgical volume and mortality in the field of cardiac surgery there remains a paucity of data regarding the relationship between surgeon volume and outcomes following LVAD implantation.^5^, ^6^, ^7^

A relationship between institution volume and VAD outcomes has been described in the literature, with studies bearing mixed results. Shah et al.^8^ described improved outcomes at high volume centers. However, another study demonstrated improved outcomes at moderate volume centers, as compared to low or high‐volume centers.^9^ There is limited data evaluating the role that individual surgeon volume plays in VAD outcomes, with a single study demonstrating a relationship between increased surgeon volume and decreased mortality.^10^ While setting volume thresholds for both hospitals and surgeons may appear intuitive, its true consequences are debated and arbitrarily imposed volume requirements may limit access to limited and life‐saving resources.^11^

With current data it is unclear if a definite relationship between surgeon volume and VAD outcomes exists. In this study, we sought to build on the available knowledge by examining the impact of surgeon volume on VAD outcomes at a center with an established and busy multidisciplinary VAD program.

## 2 METHODS

### 2.1 Patient population

Hospital medical records were queried, and data collected for all patients who underwent primary LVAD implantation at our institution from January 1, 2013 to December 31, 2019. Patients who received a right ventricular assist device (RVAD) at the index operation were counted as one event. Those who received an isolated RVAD were not included in this study. Patients who had LVADs exchanged were not included. Patients were dichotomized into two groups, a high‐volume group, and a low‐volume group, based on the average yearly case volume of the surgeon who performed the procedure. Surgeons averaging 11 or more are categorized as high volume, and those averaging 10 or less are categorized as low volume. This cut‐off was chosen based on Centers for Medicare and Medicaid Services (CMS) guidelines and previous work by Davis et al.^10^ Institutional Review Board approval (0226‐20‐EP), with individual patient informed consent waived, was obtained to perform a retrospective review of patient data.

### 2.2 Perioperative care

The implantation procedure was conducted in a standard fashion, with minimal variation in technique between surgeons. Approach favored median sternotomy during the study period with increased use of a thoracotomy approach toward the end of the study period. The choice of device evolved over time, favoring HeartMate 2 (HM2) at the beginning of the study, and gradually including more HeartMate 3 (HM3) devices as our center participated in the MOMENTUM 3 trial, followed by preferential HM3 use after commercial availability. A limited number of HeartWare devices were used for patients with small stature. The choice of surgeon was based on availability with low‐volume surgeons implanting when a high‐volume surgeon was not available, or when the case was urgent/emergent. For the early part of the study period there was a single surgeon (surgeon 1) performing the bulk of VAD implants, in the final year of the study period an additional surgeon (surgeon 2) joined the practice as high‐volume.

Cases were performed by a single surgeon; high‐volume surgeons did not assist low‐volume surgeons in their operations and vice‐versa. Our institution does not offer a transplant or advanced cardiac surgery fellowship, traditional cardiothoracic surgery trainees infrequently participated in these cases. Preoperative and postoperative management was conducted by a multidisciplinary group including heart failure cardiologists, critical care physicians, cardiothoracic surgery providers, LVAD clinicians, and clinical pharmacists. The perioperative care providers were common between the low and high‐volume groups, and postoperative management was conducted in a similar manner. Perioperative care protocols are described in Supporting Information: document 1.

### 2.3 Outcomes of interest

The primary outcome assessed was survival to hospital discharge. Other outcomes were 1‐year survival, postoperative stroke, right‐ventricle failure, pump thrombosis, driveline infection, major postoperative bleeding, bleeding requiring reoperation, GI bleeding, respiratory failure, hepatic dysfunction, and renal failure. Outcomes were defined in accordance with standard INTERMACS definitions.^12^

### 2.4 Statistical analysis

Continuous variables are presented as median with 25th and 75th percentile intervals. Categorical variables are reported as counts and percentages. *χ* ^2^ test or Fisher's exact test were used to evaluate the difference in categorical characteristics. Continuous variables comparisons were performed using the Mann−Whitney *U* test. Estimates of overall survival and freedom from adverse events were determined based on the Kaplan−Meier method, and the log rank test was used for comparisons. Survival was defined from date of LVAD implantation until date of death or last day of follow‐up. Recipients were censored at the time of transplantation, explantation, or defunctionalization for recovery. The linearized rate for each adverse event was calculated as total number of observed events divided by total patient‐months of follow‐up and expressed as episode/100 patient‐months. Rates of adverse events were compared between the two groups using Poisson regression.

To account for possible selection bias between the high and low‐volume groups, propensity score matching (PSM) was performed. Propensity scores were calculated by a logistic regression model that included 22 select preoperative and baseline variables known to be associated with outcomes. Two‐to‐one matching without replacement was performed using a caliper setting of 0.15. Two high‐volume controls were matched to 1 low‐volume case. The mean distance in propensity score between matched pairs was 0.01. Overall, 37 low volume patients were matched to 74 high volume patients, 82% of all possible matches. Selected matches were drawn from the entirety of the distribution. Covariate distribution was found to be well complemented after matching.

Univariate and Cox proportionate hazard multivariate analyses were used to examine the associations of baseline demographic and clinical characteristics (including the case‐volume of the surgeon) with survival. The entire study cohort (nonpropensity matched) was used to construct these models. Preselection of variables was performed by identifying variables in univariate analysis with *p* < .10, which were then entered in the multivariate model using forward stepwise selection. A *p *< .05 is considered significant. The statistical analyses were performed with IBM SPSS Statistics, version 26 (IBM Corporation).

## 3 RESULTS

### 3.1 Patient characteristics

**Figure 1:**
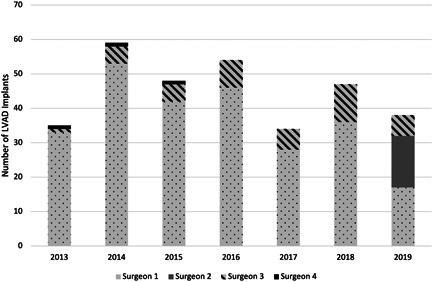
Annual case volume by surgeon, LVAD, left ventricular assist device.

**Figure 2:**
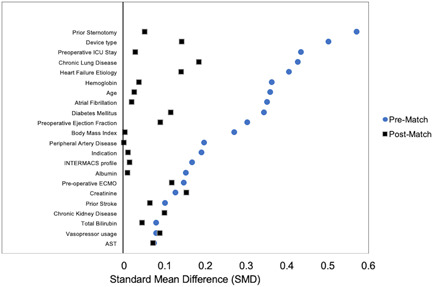
Covariate balance between matched and unmatched groups

During the study period, 315 patients underwent primary LVAD implantation; 45 had operations performed by a low‐volume surgeon; and 270 were performed by a high‐volume surgeon. Individual surgeon volume per year is demonstrated in Figure 1. Two surgeons fell in the low‐volume group (surgeons 3, 4) and two in the high‐volume group (surgeons 1, 2) with a single surgeon (surgeon 1) being the primary implanter. An average case volume of 11 or more seemed to adequately distinguish between the operators. Baseline characteristics are shown in Table 1. Patients in the high‐volume group tended to be older (median age 60 vs. 56; *p* = .02), more likely to have diabetes (40% vs. 24.4%; *p* = .05), atrial fibrillation (37.8% vs. 22.2%; *p* = .04), and a prior sternotomy (36.7% vs. 13.3%; *p* = .002). Patients in the low volume group had a higher incidence of chronic lung disease (44.1% vs. 24.1%; *p* = .004), and lower preoperative hemoglobin (10.7 vs. 11.5; *p* = .02). Choice of device varied significantly between the unmatched groups with those in the low‐volume group more likely to receive a HeartMate II (55.9% vs. 35.6%), and less likely to receive a HeartMate III device (35.2% vs. 46.7%) (*p* = .005). The baseline characteristics between the two groups were well complemented after propensity matching. Pre and postmatch distribution of covariates used to estimate the propensity score are demonstrated in Figure 2.

**Table 1:** Baseline demographics and patient characteristics^a^

Variable	Low volume unmatched (*n* = 45)	High volume unmatched (*n* = 270)	Prematch *p *value	Low volume matched (*n* = 37)	High volume matched (*n* = 74)	Postmatch *p *value
Age (years)	56 (49−62)	60 (51−67)	.02	56 (49−63)	56.5 (47−63.2)	.99
Male	33 (73.3)	207 (76.7)	.63	28 (75.7)	59 (79.7)	.63
Race, Caucasian	34 (75.6)	235 (87.0)	.18	29 (78.4)	61 (82.4)	.56
Body mass index	27.4 (23.1−33.2)	29.75 (25.2−33.7)	.07	27.5 (23.1−34.3)	27.6 (23.3−32.6)	.94
Peripheral artery disease	2 (4.4)	25 (9.3)	.39	1 (2.7)	2 (2.7)	>.99
Chronic kidney disease	20 (44.4)	106 (39.3)	.51	15 (40.5)	27 (36.5)	.68
Chronic lung disease	20 (44.4)	65 (24.1)	.004	15 (40.5)	24 (32.4)	.39
Prior stroke	5 (11.1)	21 (7.8)	.39	4 (10.8)	7 (9.5)	>.99
Diabetes mellitus	11 (24.4)	108 (40)	.05	10 (27)	16 (21.6)	.53
Atrial fibrillation	10 (22.2)	102 (37.8)	.04	8 (21.6)	17 (23)	.87
Prior sternotomy	6 (13.3)	99 (36.7)	.002	6 (16.2)	13 (17.6)	>.99
Albumin	3.3 (2.9−3.7)	3.4 (3−3.7)	.34	3.3 (2.9−3.7)	3.3 (2.9−3.7)	.99
Creatinine	1.25 (0.9−1.6)	1.19 (0.9−1.53)	.97	1.2 (0.9−1.6)	1.2 (0.9−1.6)	.78
INR	1.2 (1.1−1.4)	1.2 (1.1−1.3)	.34	1.2 (1.1−1.4)	1.2 (1.1−1.3)	.64
AST	32 (20−54)	31 (20−57)	.79	34 (23−59)	37 (22−63)	.52
ALT	30 (17−66)	30 (18−56)	.76	33 (17−66)	34 (17−77)	.83
Sodium	136 (133−138)	136 (133−138)	.80	136 (134−138)	136 (132−138)	.40
Platelets	205 (151−258)	182 (137−240)	.13	205 (151−245)	194 (129−245)	.38
Bilirubin	1.2 (0.7−1.8)	1.1 (0.7−1.6)	.47	1.2 (0.7−1.8)	1.0 (0.8−1.6)	.39
Hemoglobin	10.7 (9.1−12.1)	11.5 (99−12.9)	.02	10.8 (9.1−12.4)	10.7 (9.4−12.5)	.74
Hematocrit	32.9 (28.1−37.2)	35 (30.4−39.4)	.05	33.3 (28.3−38.7)	33.4 (28.9−38.6)	.81
Preop ICU stay	26 (57.7)	107 (39.6)	.02	20 (54)	40 (54)	>.99
Pre‐op ICU duration (days)	4.5 (2−9)	4 (2−7)	.26	1 (0−4.5)	1 (0−5)	.93
Vasopressor or inotrope usage	33 (73.3)	185 (68.5)	.52	28 (75.7)	53 (71.6)	.65
Ejection fraction	15 (10−20)	20 (15−25)	.07	15 (10−20)	15 (15−20)	.66
Right atrial pressure	12 (7−17)	12 (7−17)	.86	11.5 (6.7−18.2)	14 (7−18)	.39
Cardiac index	2.0 (1.6−2.4)	1.9 (1.6−2.3)	.37	2.02 (1.6−2.4)	1.8 (1.6−2.1)	.08
PVR	2.5 (2−4)	3 (2−4)	.94	2.4 (2−4)	3 (2−3.9)	.91
ECMO support	7 (15.6)	29 (10.7)	.35	4 (10.8)	11 (14.9)	.56
Other mechanical support	17 (37.8)	84 (31.1)	.37	15 (40.5)	29 (39.2)	.89
Mechanical ventilation	3 (6.7)	36 (13.3)	.21	2 (5.4)	11 (14.9)	.21
Intermacs profile			.22			.77
1	5 (11.1)	41 (15.2)		5 (13.5)	14 (18.9)	
2	19 (42.2)	69 (25.6)		16 (43.2)	25 (33.8)	
3	14 (31.1)	98 (36.3)		10 (27)	21 (28.4)	
4+	7 (15.6)	62 (22.9)		6 (16.2)	14 (18.9)	
Heart failure etiology			.04			.49
Ischemic	17 (37.8)	155 (57.4)		14 (37.8)	33 (44.6)	
Nonischemic	28 (62.2)	114 (42.2)		23 (62.2)	41 (55.4)	
Intention to treat			.14			.38
Destination	31 (68.9)	171 (63.3)		24 (64.9)	51 (68.9)	
Bridge to transplant	10 (22.2)	89 (33)		9 (24.3)	20 (27)	
Bridge to recovery	4 (8.9)	0		4 (10.8)	0	
Device type			.005			.53
HeartMate 2	16 (35.6)	160 (59.3)		15 (40.5)	29 (39.2)	
HeartMate 3	21 (46.7)	90 (33.3)		18 (48.6)	31 (41.9)	
HVAD	8 (17.8)	20 (7.4)		4 (10.8)	14 (18.9)	

Abbreviations: AST, aspartate aminotransferase; ALT, alanine transaminase; CPB, cardiopulmonary bypass; ECMO, extracorporeal membrane oxygenation; HVAD, heartware HVAD; INR, international normalized ratio; PTT, partial thromboplastin time; PVR, pulmonary vascular resistance; RVAD, right ventricular assist device.

^a^ Expressed as median (interquartile range) or count (percentage).

### 3.2 Survival

**Figure 3:**
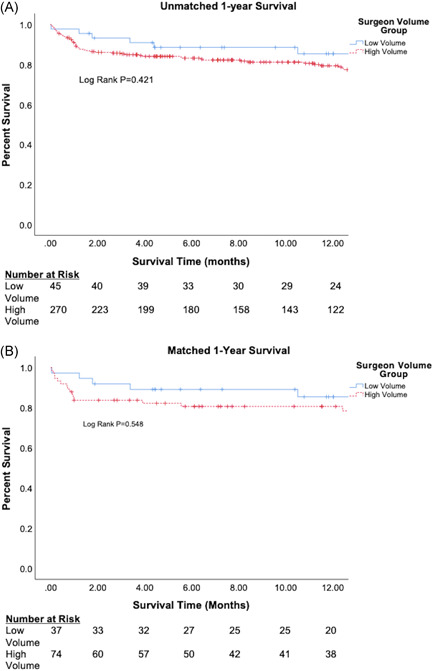
(A) One‐year survival of unmatched patients, (B) One‐year survival of matched patients

In the unmatched cohort, discharge survival was equivalent between the low and high‐volume groups (93.3% vs. 86.7%; *p* = .22). Survival at 1 year was also equivalent (85.4% vs. 79.0%; *p* = .42; Figure 3A). In the PSM cohort, there was no difference in survival to hospital discharge between the groups (91.9% low volume vs. 83.3% high volume; *p* = .22). Survival at 1 year did not differ significantly (85.4% low volume vs. 80.6% high volume; *p* = .55; Figure 3B). To analyze outcomes amongst the sickest patients, we conducted a subgroup analysis of those patients who had an INTERMACS profile of 1 (critical cardiogenic shock) or 2 (progressive hemodynamic decline despite inotropic support). In the PSM cohort, 21 low‐volume patients (56.7%) and 37 high‐volume patients (50%) met these criteria. Discharge survival in these INTERMACS I/II patients did not differ between surgeon volume groups (90.5% low volume vs. 86.5% high volume; *p* > .99).

### 3.3 Adverse events

There was no significant difference in adverse events between the propensity matched groups (Table 2). GI bleeding trended towards significance (*p* = .085) with a rate of 4.55 per 100 patient months in the low‐volume group, and 2.95 per 100 patient months in the high‐volume group.

**Table 2:** Outcomes of matched pairs

Outcomes (%)	Low‐volume matched		High‐volume matched		*p *value
Survival to discharge	34 (91.9)		60 (83.3)		.22
Reoperation for bleeding	4 (10.8)		10 (13.5)		.77
Major postoperative bleeding	17 (45.9)		36 (48.6)		.78
RV failure	12 (32.4)		22 (29.7)		.77
Respiratory failure	8 (21.6)		18 (24.3)		.75
Hepatic failure	3 (8.1)		6 (8.1)		>.99
Renal failure	10 (27)		19 (25.7)		.88

**Outcomes**	**Low‐volume occurrences**	**Low‐volume rate** a	**High‐volume occurrences**	**High‐volume rate** a	***p *** **value** b
GI bleed	28	4.55	35	2.95	.085
CerebVA	4	0.65	15	1.26	.21
Pump thrombosis	8	1.30	11	0.92	.50
Major infection	20	3.25	56	4.72	.18
Driveline infection	6	0.97	13	1.09	.80

Abbreviations: CVA, cerebrovascular accident; GI, gastrointestinal.

^a^ Expressed as events/100 patient‐months.

^b^ *p *Value calculated with Poisson regression.

### 3.4 Predictors of short‐term mortality

Logistic regression analysis was performed to identify predictors of hospital mortality. Of the variables included in univariate analysis, 16 met the criteria for significance and were thus included in multivariate analysis (Table 3). In the multivariate model, older age (odds ratio [OR]: 1.038; 95% confidence interval [CI]: 1.02−1.06; *p* = .001), requiring extracorporeal membrane oxygenation (veno‐venous typically configured as an RVAD) support at the end of the procedure (OR: 9.523; 95% CI: 2.07−43.47; *p* = .004), and increasing international normalized ratio (INR) (OR: 1.072; 95% CI: 1.02−1.13; *p* = .001) were independently associated with increased hospital mortality. Surgeon volume group was not predictive of mortality on multivariate analysis (OR: 0.628; *p* = .428).

**Table 3:** Univariate and multivariate analysis of survival to discharge

Univariate regression	OR (95% CI)	*p *value
Age	0.94 (0.91−0.98)	.002
ECMO support pre‐op	0.27 (0.12−0.62)	.004
Mechanical ventilation pre‐op	0.18 (0.08−0.41)	<.001
Albumin	2.83 (1.48−5.42)	.003
INR	0.04 (0.01−0.15)	<.001
AST	0.99 (0.97−0.99)	.02
ALT	0.99 (0.99−0.99)	.006
Platelet count	1.008 (1.003−1.01)	.001
Total bilirubin	0.792 (0.66−0.95)	.04
Hemoglobin	1.29 (1.07−1.55)	.007
Hematocrit	1.09 (1.02−1.15)	.005
Right atrial pressure	0.93 (0.87−0.99)	.03
Pre‐op ICU stay	0.92 (0.86−0.99)	.03
INTERMACS profile	1.73 (1.20−2.48)	.003
CPB time	0.99 (0.98−0.99)	.02
Surgeon volume group	2.14 (0.63−7.27)	.21
Multivariable logistic regression		
Age	1.038 (1.02−1.06)	.001
ECMO after procedure	9.523 (2.07−43.47)	.004
Elevated INR	1.072 (1.02−1.13)	.001

*Note*: Baseline variables with a *p *< .1 in univariate analysis were included into the multivariate model.

Abbreviations: AST, aspartate aminotransferase; ALT, alanine transaminase; CI, confidence interval; CPB, cardiopulmonary bypass; ECMO, extracorporeal membrane oxygenation; INR, international normalized ratio; OR, odds ratio.

### 3.5 Predictors of long‐term mortality

Univariate and Cox proportional‐hazards multivariate analysis models were constructed to identify predictors of 1‐year survival from baseline variables. Univariate analysis outcomes are listed in Table 4. In the multivariate model, predictors of mortality in the first year included: older age (hazards ratio [HR]: 1.061, 95% CI: 1.03−1.09; *p* < .001), prior sternotomy (HR: 1.991, 95% CI: 1.15−3.45; *p* = .01), increasing INR (HR: 4.748, 95% CI: 2.37−9.51; *p* < .001), increasing AST (HR: 1.001, 95% CI: 1.001−1.001; *p* < .001), increasing bilirubin (HR: 1.081, 95% CI: 1.02−1.15; *p* = .01), and preoperative mechanical ventilation (HR: 2.662, 95% C:I 1.35−5.25; *p* = .005). Individual surgeon volume was not an independent predictor of 1‐year survival (HR: 0.154, *p* = .69).

**Table 4:** One‐year survival univariate and Cox proportionate hazards multivariate analysis

Univariate model	HR (95% CI)	*p *value
Age	1.03 (1.01−1.05)	.001
PAD	2.25 (1.21−4.19)	.01
Prior sternotomy	2.27 (1.45−3.54)	<.001
Ischemic etiology	2.11 (1.29−3.46)	.003
BTT	0.60 (0.34−1.07)	.08
Heartmate 3	0.42 (0.19−0.94)	.03
ECMO support pre‐op	1.99 (1.12−3.56)	.02
Non‐ECMO mechanical support	1.55 (0.98−2.44)	.06
Mechanical ventilation pre‐op	2.37 (1.37−4.12)	.002
INTERMACS profile	0.72 (0.55−0.94)	.03
Albumin	0.46 (0.31−069).	<.001
Creatinine	1.67 (1.17−2.40)	.005
INR	4.07 (2.14−7.74)	<.001
Platelet	0.99 (0.99−0.99)	.001
Hemoglobin	0.79 (0.70−0.89)	<.001
Hematocrit	0.92 (0.89−0.96)	<.001
AST	1.001 (1.000−1.001)	<.001
ALT	1.001 (1.001−1.002)	<.001
Total bilirubin	1.096 (1.04−1.16)	.001
Pre‐op ICU stay duration	1.05 (1.01−1.09)	.02
CPB time	1.004 (1.001−1.008)	.03
RVAD at procedure	2.18 (1.29−3.68)	.003
ECMO at end of procedure	1.96 (1.11−3.44)	.02
Low volume surgeon	0.76 (0.39−1.48)	.42
Cox multivariate model		
Age	1.061 (1.03−1.09)	<.001
Prior sternotomy	1.991 (1.15−3.45)	.01
INR	4.748 (2.37−9.51)	<.001
AST	1.001 (1.001−1.001)	<.001
Total bilirubin	1.081 (1.02−1.15)	.01
Preoperative mechanical ventilation	2.662 (1.35−5.25)	.005

*Note*: Baseline variables with a *p *< .1 in univariate analysis were included into the multivariate model.

Abbreviations: AST, aspartate aminotransferase; ALT, alanine transaminase; BTT, bridge to transplant; CPB, cardiopulmonary bypass; ECMO, extracorporeal membrane oxygenation; HR, hazards ratio; INR, international normalized ratio; PAD, peripheral arterial disease; RVAD, right ventricular assist device.

## 4 DISCUSSION

We investigated the impact of surgeon volume on the outcomes of patients undergoing LVAD implantation and did not identify a significant impact of surgeon volume upon outcomes. Higher surgeon volume has been associated with improved outcomes in a variety of noncardiac surgical procedures as well as cardiac surgeries, including coronary artery bypass and aortic valve replacement.^5^, ^6^, ^7^

In the field of ventricular assist device therapy, several studies have examined the relationship between center volume and outcomes. Cowger et al.^9^ demonstrated that patients receiving an LVAD at moderate volume centers, performing 31−50 procedures per year, experienced superior outcomes to those at low (<10 implants per year) or high‐volume centers (>50 implants per year). Shah et al.^8^ concluded that centers with higher LVAD volumes experienced a significant decrease in hospital mortality as well as a reduced length of stay. The authors established a threshold of 20 procedures per year that was necessary for a center to achieve in‐hospital mortality rates less than 10%.^8^ Similar results have been obersved in other studies with the threshold for volume varying slightly between studies. Lietz et al.^13^ observed improved 1‐year survival in centers performing greater than 9 procedures per year. Feldman et al.^14^ found decreased mortality particularly in centers performing 10 or greater LVADs per year. A final paper identified that centers performing greater than 15 implants per year experienced improved 90 day and overall mortality rates.^15^ Additional center considerations, such as the presence of an associated transplant program, may also contribute to LVAD outcomes.^16^

Although these studies established a relationship between center volume and outcomes the impact of individual surgeon volume within the center remained unclear. Davis et al.^10^ concluded that surgeon volume was, in fact, the primary determinant of in‐hospital mortality following VAD implantation. The authors noted a particularly significant increase in mortality as volumes fell below 12 procedures per surgeon per year. In contrast to most other literature on the topic, they concluded that annual center volume was not a significant determinant of risk adjusted in‐hospital mortality.

Our results demonstrated no significant difference in the primary endpoint of hospital mortality. Secondary outcomes, including survival at 1‐year, as well as adverse events, were also similar between the two groups. Over the study period, four surgeons performed adult LVAD implantations at our institution. Two surgeons averaged 10 or less procedures per year and were categorized as low volume, the other two surgeons were “high volume.” Despite not meeting the threshold of 12 procedures per year proposed by Davis et al.,^10^ low‐volume surgeons at our institution experienced similar outcomes to their high‐volume colleagues. An obvious concern is that higher‐risk patients were disproportionately directed to high‐volume surgeons thus unbalancing the groups. This is not the practice at our institution reflected by the equivalent distribution of INTERMACS profiles between the volume groups and between individual surgeons, and the lack of association between surgeon volume and outcomes on multivariate analysis. Nevertheless, there were differences in baseline characteristics in the unmatched groups, to control for this we performed propensity matching which yielded similar results to the main cohort. In addition, the outcomes achieved by high and low volume surgeons were similar even in the patients with the highest acuity (INTERMACS I and II). High‐volume surgeons did not assist low‐volume surgeons in their operations, eliminating this potential confounder. Notably surgeons implanting VADs in this study are experienced cardiac surgeons or possess advanced training in transplantation and mechanical circulatory support. Surgeons 1−3 have advanced training in mechanical support, surgeons 2 and 3 had just started independent practice during the study period. Surgeon 4 lacks this advanced training but had greater than 10 years in practice at the time of his implants. All surgeons are involved in the care of patients with heart failure and mechanical support to varying degrees.

It is hypothesized that larger volume centers accumulate experience, develop algorithmic protocols, and gather resources that enable improved outcomes through patient optimization, prevention and earlier detection of complications, and their management. Such multidisciplinary management strategies have been shown to shorten length of stay, improve rates of adverse events, and even decrease mortality.^17^ Similar strategies are employed at our institution (Supporting Information: document 1) with preoperative optimization followed by standard surgical implantation and postoperative management‐initially in the cardiovascular ICU, with a multidisciplinary team including intensivists, the surgeon, and the heart‐failure cardiologist. After the initial period of acuity, care is transitioned primarily to the heart failure cardiologist. With this combination of shared care, teamwork, and standardized management it is possible that individual surgeon volume does not significantly impact patient outcome. Instead, predictors of mortality in our study reiterated those previously described and included patient factors that are surrogates for acuity.^2^, ^3^, ^4^, ^15^

There are several limitations to this study. This was a retrospective review of a single institutions experience consisting of a limited number of surgeons. Intrinsic to this study design, it is possible that variables not identified or accounted for could contribute to the results of the study. Additionally, PSM is an imperfect technique and differences between groups may remain despite matching. The temporal trends in volume between the surgeons led to unintentional differences in type of device placed, with low volume surgeons participating to a greater degree toward the beginning of the study and therefore implanting a greater percentage of HM2 devices compared to their high volume colleagues. If anything, this should have biased results against the low volume surgeons given the improved results with the newer generation of devices.^18^ Our institution has an established LVAD program in conjunction with a cardiac transplantation program such that the results of the study may not be readily generalized to low‐volume or inexperienced LVAD centers. Similarly, the results of this study may be most applicable to surgeons with comparable experience, and less so to individuals with a lack of advanced training, or minimal heart failure involvement. Nevertheless, our experience is valuable as it places the relationship between surgeon volume and VAD outcomes within the context of other patient factors and center experience demonstrating that these other factors may be more critical to outcomes.

Current VAD volume standards from the CMS dictate that a center must have at least one surgeon with 10 VAD implants over a 3‐year period, and VAD activity in the last 12 months with no criteria for additional surgeons.^19^ The results of our study, alongside previous studies, on the VAD‐volume relationship have broader implications for dispersal of VAD resources in the country. Presently, 188 centers of varying experience are certified for VAD implantation in the United States.^20^ Previous authors have forcefully advocated for standards in surgery based upon both surgeon and hospital volume.^5^ Current data may support the use of center volume as a guide for maintaining quality and outcomes but does not conclusively support surgeon volume as a similar guide. Clearly there is an interaction between surgeon experience, center volume, and patient factors that leads to optimal outcomes, but hard cut‐offs remain elusive. We caution for the measurement of hard outcomes over volume‐based surrogates of outcomes. Patient acuity should be utilized to guide referral of patients to centers with greater experience.

## 5 CONCLUSIONS

In this study there was no association between surgeon volume and survival or adverse events after LVAD implantation. These results need to be interpreted cautiously as they represent the experience of a single busy VAD program with a limited number of experienced surgeons. The results suggest that, in a multidisciplinary team, low‐volume VAD surgeons can achieve comparable outcomes to their high‐volume colleagues. The impact of surgeon volume warrants further examination as it has implications for volume‐based guidelines.

## CONFLICT OF INTEREST

The authors declare no conflict of interest.

## ETHICS STATEMENT

University of Nebraska Institutional review board approval with individual patient consent waived (0226‐20‐EP).

## Supporting information

Supporting information.

## References

[1] Molina EJ , Shah P , Kiernan MS , et al. The Society of Thoracic Surgeons INTERMACS 2020 annual report. Ann Thorac Surg. 2021;111(3):778‐792. 33465365 10.1016/j.athoracsur.2020.12.038

[2] Birati EY , Hanff TC , Maldonado D , et al. Predicting long term outcome in patients treated with continuous flow left ventricular assist device: the Penn‐Columbia risk score. J Am Heart Assoc. 2018;7(6):e006408. 29514805 10.1161/JAHA.117.006408 PMC5907534

[3] Akin S , Soliman O , de By TMMH , et al. Causes and predictors of early mortality in patients treated with left ventricular assist device implantation in the European Registry of Mechanical Circulatory Support (EUROMACS). Intensive Care Med. 2020;46:1349‐1360. 32016536 10.1007/s00134-020-05939-1 PMC7334284

[4] Gonuguntla K , Patil S , Cowden RG , et al. Predictors of in‐hospital mortality in patients with left ventricular assist device. Ir J Med Sci. 2020;189:1275‐1281. 32388673 10.1007/s11845-020-02246-y

[5] Birkmeyer JD , Stukel TA , Siewers AE , Goodney PP , Wennberg DE , Lucas FL . Surgeon volume and operative mortality in the United States. N Engl J Med. 2003;349(22):2117‐2127. 14645640 10.1056/NEJMsa035205

[6] Dewey TM , Herbert MA , Ryan WH , et al. Influence of surgeon volume on outcomes with aortic valve replacement. Ann Thorac Surg. 2012;93(4):1107‐1113. 22226491 10.1016/j.athoracsur.2011.09.064

[7] Bashir M , Harky A , Fok M , et al. Acute type A aortic dissection in the United Kingdom: surgeon volume‐outcome relation. J Thorac Cardiovasc Surg. 2017;154(2):398‐406. 28291608 10.1016/j.jtcvs.2017.02.015

[8] Shah N , Chothani A , Agarwal V , et al. Impact of annual hospital volume on outcomes after left ventricular assist device (LVAD) implantation in the contemporary era. J Card Fail. 2016;22(3):232‐237. 26547012 10.1016/j.cardfail.2015.10.016

[9] Cowger JA , Stulak JM , Shah P , et al. Impact of center left ventricular assist device volume on outcomes after implantation. J Am Coll Cardiol HF. 2017;5(10):691‐699. 10.1016/j.jchf.2017.05.011 PMC8453659 28888521

[10] Davis KF , Hohmann SF , Doukky R , Levine D , Johnson T . The impact of hospital and surgeon volume on in‐hospital mortality of ventricular assist device recipients. J Card Fail. 2016;22(3):226‐231. 26505811 10.1016/j.cardfail.2015.10.012

[11] Schwartz DM , Fong ZV , Warshaw AL , Zinner MJ , Chang DC . The hidden consequences of the volume pledge: “no patient left behind”? Ann Surg. 2017;265(2):273‐274. 27280503 10.1097/SLA.0000000000001833

[12] UAB . INTERMACS Appendices‐Interagency Registry for Mechanically Assisted Circulatory Support. Appendix O: INTERMACS profiles of advanced heart failure. 2021. Accessed May 28, 2021. https://www.uab.edu/medicine/intermacs/images/protocol_4.0/protocol_4.0_MoP/Appendix_O_Intermacs_Patient_Profile_at_time_of_implant.pdf

[13] Lietz K , Long JW , Kfoury AG , et al. Impact of center volume on outcomes of left ventricular assist device implantation as destination therapy: analysis of the Thoratec HeartMate Registry, 1998−2005. Circ Heart Fail. 2009;2(1):3‐10. 19808309 10.1161/CIRCHEARTFAILURE.108.796128

[14] Feldman K , Doukky R , Johnson T , et al. Hospital volume of LVAD procedures determines patient outcome. Cir: Cardiovas Qual Outcomes. 2013;6:A289.

[15] Cowger J , Sundareswaran K , Rogers JG , et al. Heart failure predicting survival in patients receiving continuous flow left ventricular assist devices: the HeartMate II risk score. J Am Coll Cardiol. 2013;61(3):313‐321. 23265328 10.1016/j.jacc.2012.09.055

[16] Brinkley DM , DeNofrio D , Ruthazer R , et al. Outcomes after continuous‐flow left ventricular device implantation as destination therapy at transplant versus nontransplant centers. Circ Heart Fail. 2018;11(3):e004384. 29540471 10.1161/CIRCHEARTFAILURE.117.004384

[17] Jorde UP , Shah AM , Sims DB , et al. Continuous‐flow left ventricular assist device survival improves with multidisciplinary approach. Ann Thorac Surg. 2019;108(2):508‐516. 30853587 10.1016/j.athoracsur.2019.01.063

[18] Mehra MR , Naka Y , Uriel N , et al. A fully magnetically levitated circulatory pump for advanced heart failure. N Engl J Med. 2017;376(5):440‐450. 10.1056/NEJMoa1610426 27959709

[19] CMS. Decision memo for artificial hearts and related devices, including ventricular assist devices for bridge‐to‐transplant and destination therapy. 2021. Accessed June 1, 2021. https://www.cms.gov/medicare-coverage-database/details/nca-decision

[20] CMS . VAD destination therapy facilities. 2021. Accessed June 12, 2021. https://www.cms.gov/Medicare/Medicare-General-Information/MedicareApprovedFacilitie/VAD-Destination-Therapy-Facilities

