# Supplementary material for: In a large‐volume multidisciplinary setting individual surgeon volume does not impact LVAD outcomes
Source: J Card Surg. 2022 Jul 21;37(10):3290–9. doi: 10.1111/jocs.16783 (PMC9542019; doi:10.1111/jocs.16783)
Supplement: Supplementary file 1 — Supporting information. [file JOCS-37-3290-s001.docx]

Bundle for RV failure prevention in LVAD recipients

**Pre-op**

-Aggressive diuresis to achieve CVP < 15 and clinical euvolemia whenever possible.

-Correct coagulopathy

**Intra-op**

-Continuation of low tidal volume ventilation during cardiopulmonary bypass.

-Pacing to keep HR at least 80 or more.

-Inhaled pulmonary vasodilators to be initiated before weaning from bypass.

-Minimize pulmonary vasoconstriction- aggressive prevention/correction of hypercapnia/hypoxia, reversal of acidosis; preference for vasopressin over levophed.

-Inotropic infusions to be initiated before coming off bypass.

-Aggressive correction of coagulopathy. Consider pre-emptive 2 units plts after weaning from bypass. Consider checking fibrinogen while on bypass and giving cryo after bypass if the fibrinogen < 220. Consider use of TEG.

**Post-op**

-Early extubation as soon as meets usual criteria (ie stable hemodynamics, no significant acidosis/hypercapnia/hypoxia)

-Minimize pulmonary vasoconstriction. Aggressive prevention/correction of hypercapnia/hypoxia, reversal of acidosis. Preference for vasopressin over levophed.

-Minimize iv fluids if CVP high, diurese to keep CVP < 15, wean inotropes/pulmonary vasodilators to keep CVP < 15.

-Manage afterload to keep MAP 70-90. This may require vasodilators (iv nitro, nicardipine, oral ACEi, hydralazine).

-Adjust LVAD flows starting POD1 to keep PCWP < 20.

- Consider PA cath (Swan Ganz) removal when on low dose single inotrope with stable PCWP < 20, CVP < 15 and CI > 2.2.

-Once clinically euvolemic record dry weight and adjust diuretics to keep patient at or below dry weight.
